# Intense Cold as a Local Anæsthetic

**Published:** 1855-09

**Authors:** D. W. Hammond

**Affiliations:** Macon, Georgia


ARTICLE V.—*Intense Cold as a Local Anæsthetic.* By D. W. HAMMOND, M. D., Macon, Georgia.

CASE 1.—Mr. Jacob Young, a Clerk of the Superior Court of Irwinville, Irwin County, Ga., came to this city on the 14th of May last, and consulted me in reference to a large tumor located near, and to the right of, the lumbar vertebræ, about two and a half inches above the posterior crest of the ilium, and lying upon the aponeurosis of the latissimus dorsi muscle.

On consultation with Dr. Boon, its removal was determined upon at once. In consequence of the extreme pain experienced from a previous *unsuccessful* operation, and having objections to taking chloroform, we determined to use the refrigerating mixture. Equal parts of pounded ice and salt were prepared, and enclosed in a towel, and applied over the tumor. In less than four minutes it was completely congealed, together with the surrounding integuments and subcutaneous areolar tissue. The tumor now presented a blanched appearance, and diminished in size to one-third of its natural dimensions; in fact, it was so perfectly solidified and friable, that I was apprehensive, in dissecting off the investing integuments, that they might crumble. To prevent this, I passed a wet sponge over the surface in order to lessen the intensity of the congelation prior to the operation. With a common scalpel an incision was made from above, downwards, six inches in length, dividing the skin and subcutaneous adipose tissue down to the diseased structure. Being now entirely exposed, the upper lobe was raised by a strong tenaculum, and quite easily disengaged (together with two others) by a few touches of the knife.

The operation was comparatively a painless one. Not more than one-fourth of an ounce of blood was lost during its performance. A slight hemorrhage came on after reaction was established, but not sufficient to require the ligation of a single artery. The tumor was four inches in length, tri-lobed, and from one and a half to three inches in diameter, and of a fibrous structure.

The integuments were approximated and retained by two sutures and adhesive straps. On the third day the dressings were removed—the wound presented a healthy appearance and was healing rapidly. After two or three subsequent dressings, the wound was so nearly healed that Mr. Young left for his home, much gratified with the prospects of a speedy and radical cure. Not having heard from him since he left, I presume the operation was successful and entirely satisfactory.

CASE 2.—I received a note from Professor Smith, President of the Wesleyan Female College, requesting me to see Miss Yopp, one of the young ladies of the graduating class at our last commencement. On examination, I found a small encysted *Atheromatous* tumor, about the size of a filbert, situated in the palm of the hand, immediately under the skin, and a little external to the centre of the palmar fascia. The tumor, although small, destroyed to considerable extent the mobility of the third and little finger, rendering it difficult to make the requisite palpations upon the keys of the piano forte.

Its removal being requested, Dr. Boon prepared the "frozen mixture," and applied it by enveloping it in the corner of a linen handkerchief. In two minutes the tumor was congealed, the skin bleached and corrugated. The skin was now severed by a slight stroke of the knife, which brought the investing cyst into view. With a small pair of curved forceps it was readily seized and pulled out. Only a few drops of blood lost. Dressed with Isinglass plaster, compress and roller bandage. The wound healed by the first intention.

